# Dual Convolutional Neural Networks With Attention Mechanisms Based Method for Predicting Disease-Related lncRNA Genes

**DOI:** 10.3389/fgene.2019.00416

**Published:** 2019-05-03

**Authors:** Ping Xuan, Yangkun Cao, Tiangang Zhang, Rui Kong, Zhaogong Zhang

**Affiliations:** ^1^School of Computer Science and Technology, Heilongjiang University, Harbin, China; ^2^School of Mathematical Science, Heilongjiang University, Harbin, China; ^3^Department of Pancreatic and Biliary Surgery, The First Affiliated Hospital of Harbin Medical University, Harbin, China

**Keywords:** lncRNA-disease prediction, dual convolutional neural networks, attention at feature level, attention at relationship level, lncRNA-miRNA interactions

## Abstract

A lot of studies indicated that aberrant expression of long non-coding RNA genes (lncRNAs) is closely related to human diseases. Identifying disease-related lncRNAs (disease lncRNAs) is critical for understanding the pathogenesis and etiology of diseases. Most of the previous methods focus on prioritizing the potential disease lncRNAs based on shallow learning methods. The methods fail to extract the deep and complex feature representations of lncRNA-disease associations. Furthermore, nearly all the methods ignore the discriminative contributions of the similarity, association, and interaction relationships among lncRNAs, disease, and miRNAs for the association prediction. A dual convolutional neural networks with attention mechanisms based method is presented for predicting the candidate disease lncRNAs, and it is referred to as CNNLDA. CNNLDA deeply integrates the multiple source data like the lncRNA similarities, the disease similarities, the lncRNA-disease associations, the lncRNA-miRNA interactions, and the miRNA-disease associations. The diverse biological premises about lncRNAs, miRNAs, and diseases are combined to construct the feature matrix from the biological perspectives. A novel framework based on the dual convolutional neural networks is developed to learn the global and attention representations of the lncRNA-disease associations. The left part of the framework exploits the various information contained by the feature matrix to learn the global representation of lncRNA-disease associations. The different connection relationships among the lncRNA, miRNA, and disease nodes and the different features of these nodes have the discriminative contributions for the association prediction. Hence we present the attention mechanisms from the relationship level and the feature level respectively, and the right part of the framework learns the attention representation of associations. The experimental results based on the cross validation indicate that CNNLDA yields superior performance than several state-of-the-art methods. Case studies on stomach cancer, lung cancer, and colon cancer further demonstrate CNNLDA's ability to discover the potential disease lncRNAs.

## Introduction

Long non-coding RNA genes (lncRNAs) are transcripts longer than 200 nucleotides which are not translated into proteins (Reik, 2009). Accumulating evidences have indicated that lncRNAs play crucial roles in the metastasis and progression of various diseases (Prensner and Chinnaiyan, 2011; Schmitt and Chang, 2016; Hu et al., 2018). Therefore, identifying the associations between lncRNAs and diseases is important for understanding the functions of lncRNAs in the disease processes.

Predicting disease-related lncRNAs (disease lncRNAs) can screen the potential candidates for the biologists to discover the real lncRNA-disease associations with the wet-lab experiments (Chen et al., 2016a). Existing methods have been presented for prioritizing the candidate disease lncRNAs, which fall into three main categories. Methods in the first category utilize the biological information related to lncRNAs, such as the genome locations, tissue specificity and expression profile. Chen et al. and Li et al. predicted disease lncRNAs by exploiting the locations of lncRNAs and genes in the genome (Chen et al., 2013; Li et al., 2014a). However, the methods are not effective on the lncRNAs which have no adjacent genes. Liu et al. and Chen predicted the potential associations by using the lncRNA tissue specificity or lncRNA expression profile (Liu et al., 2014; Chen, 2015). The methods suffered from the limited information of tissue-specific expressions and low expression levels of lncRNAs.

Methods in the second category construct the prediction models based on machine learning for inferring the lncRNA-disease associations. A semi-supervised learning based method was proposed to predict the potential associations (Chen and Yan, 2013). On the basis of this study, Chen et al. and Huang et al. optimized the calculation of the similarities of lncRNAs and diseases (Chen et al., 2015; Huang et al., 2016). However, the methods considered the information of the lncRNA and disease spaces, and did not fuse them completely. Several methods infer the candidate lncRNAs related to a disease by random walk on the lncRNA functional similarity network or heterogeneous network composed of lncRNAs, genes and diseases (Sun et al., 2014; Chen et al., 2016b; Gu et al., 2017; Yao et al., 2017). The common and similar neighbors of two diseases (or two lncRNAs) in the lncRNA-disease bipartite network are utilized to infer the association scores between lncRNAs and diseases (Ping et al., 2018). Nevertheless, most of these methods fail to be applied to new diseases without any known related lncRNAs.

The methods in the third category integrate the multiple data sources about the proteins and miRNAs that are interacted with lncRNAs, and the drugs associated with the proteins. Zhang et al. constructed the lncRNA-protein-disease network and obtained the candidate disease lncRNAs by propagating information flow in the heterogeneous network (Zhang et al., 2017). After calculating the various lncRNA and disease similarities, LDAP used the bagging SVM classifier to uncover the potential diseases lncRNAs(Lan et al., 2017). A couple of methods established the matrix factorization based prediction models to fuse the multiple kinds of information related to the lncRNAs, diseases and proteins (Fu et al., 2017; Lu et al., 2018). However, most of the previous methods are the shallow learning methods which cannot learn the deep and complex representations of lncRNA-disease associations.

Deep learning approaches can hold the promise of much better performance (Xu et al., 2017). In our study, we propose a novel method based on dual convolutional neural networks to predict lncRNA-disease associations, which we refer to as CNNLDA. CNNLDA exploits the similarities and associations of lncRNAs and diseases, the interactions between lncRNAs and miRNAs, and the miRNA-disease associations. The feature matrix is firstly constructed based on the biological premises about lncRNAs, miRNAs, and diseases. Combining the biological premise about the cases that two lncRNAs (diseases) should be more similar can capture the relationships between the lncRNA-disease associations and the lncRNA (disease) similarities. Integrating the interactions between lncRNAs and miRNAs, and the miRNA-disease associations can capture the relationships between the lncRNAs and miRNAs interacted with each other and the lncRNA-disease associations. A new framework based on the dual convolutional neural networks is established for extracting both the global and the attention feature representations of lncRNA-disease associations. The left part of the framework is concentrated on extract features from the associations and similarities of lncRNAs and diseases. In the right part of the framework, each of features and each kind of features are assigned to different weights by applying our proposed attention mechanisms, which may discriminate their different contributions for predicting the potential disease lncRNAs. The comprehensive cross-validation experiments confirm that CNNLDA outperforms several state-of-the-art methods for predicting candidate disease lncRNAs. Moreover, case studies on 3 diseases indicate that CNNLDA is able to discover potential association candidates that are supported by the corresponding databases and literature.

## Materials and Methods

### Datasets for Disease lncRNA Prediction

The lncRNA-disease associations, the lncRNA-miRNA interactions, and the miRNA-disease associations are obtained from the previous work on prediction of the lncRNA-disease associations (Fu et al., 2017). The 2687 lncRNA-disease associations are originally extracted from the databases LncRNADisease (Chen et al., 2013) and Lnc2Cancer (Ning et al., 2016) that contains the experimentally confirmed lncRNA-disease associations, and the database GeneRIF (Lu et al., 2006) that records the lncRNA functional description. The 1002 lncRNA-miRNA interactions are extracted from database starBase (Li et al., 2014b) which includes the interaction information between multiple kinds of RNAs. The disease semantic similarities are obtained from DincRNA (Cheng et al., 2018) that are used by us to calculate the lncRNA similarities based on their associated diseases. The 5218 verified miRNA-disease associations by experiment are obtained from the human miRNA-disease database HMDD (Li et al., 2013). All of these associations and interactions cover 240 lncRNAs, 402 diseases, and 495 miRNAs.

### Calculation and Representation of Multiple Kinds of Data

#### Representation of the lncRNA-Disease Associations and miRNA-Disease Associations

**Figure 1:**
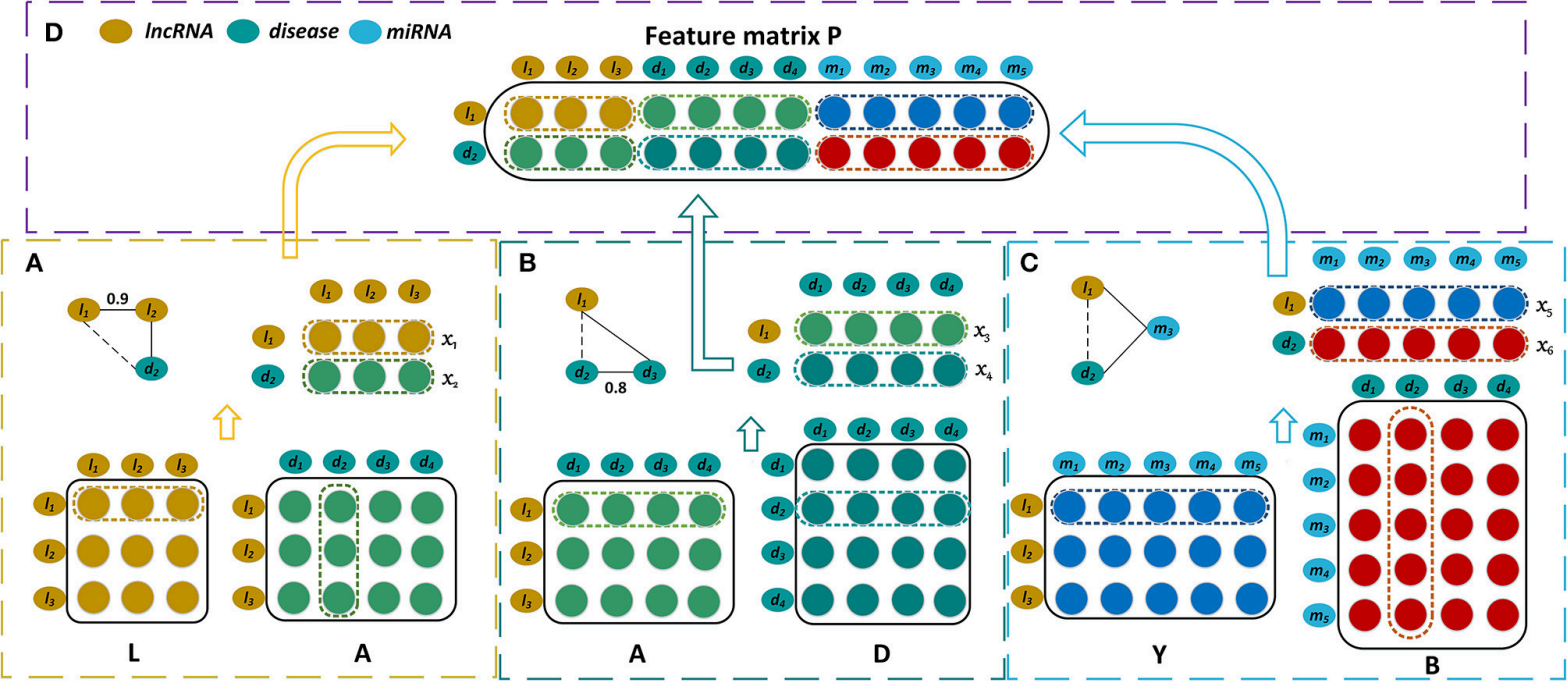
Construction of the feature matrix of lncRNA *l*_1_ and disease *d*_2_. **(A)** Construct the first part of feature matrix by integrating the lncRNA similarities and the lncRNA-disease associations. **(B)** Construct the second part by incorporating the lncRNA-disease associations and the disease similarities. **(C)** Construct the third part by exploiting the lncRNA-miRNA interactions and the miRNA-disease associations. **(D)** Concatenate these three parts to form the feature matrix ***P***.

The bipartite graph composed of lncRNAs and diseases is constructed by the known lncRNA-disease associations (Figure 1A). We use matrix $A\epsilon {\mathbb{R}}^{n_{l}\times n_{d}}$ to represent the association case between *n*_*l*_ lncRNAs and *n*_*d*_ diseases, where *A*_*ij*_ is 1 if lncRNA *l*_*i*_ has been observed to be related to disease *d*_*j*_ or 0 otherwise. As shown in Figure 1C, the known miRNA-disease associations form the miRNA-disease bipartite graph. Matrix $B\epsilon {\mathbb{R}}^{n_{m}\times n_{d}}$ represents the associations between *n*_*m*_ miRNAs and *n*_*d*_ diseases. *B*_*ij*_ is set to 1 means there is observed association between miRNA *m*_*i*_ and disease *d*_*j*_, and it is 0 otherwise.

#### Representation of the Disease Similarities

The more similar that two diseases are, the more likely that they are associated with similar lncRNAs. Hence the disease similarities are integrated by our model for predicting disease-related lncRNAs. A disease can be represented by a directed acyclic graph (DAG) that includes all the disease terms related to the disease. If two diseases have more common disease terms, they are more similar, which is the basic idea for semantic similarity between Gene Ontology terms (Xu et al., 2013b). Wang et al. have successfully measured the similarity of two diseases based on their DAGs (Wang et al., 2010). The disease similarities are calculated by Wang's method, and they are represented by matrix $D\epsilon {\mathbb{R}}^{n_{d}\times n_{d}}$ where *D*_*ij*_ is the similarity of two diseases *d*_*i*_ and *d*_*j*_(Figure 1B).

#### Representation of the lncRNA Similarities

As the lncRNAs associated with the similar diseases are generally possible to have more similar functions, Chen et al. measured the similarity of two lncRNAs based on their associated diseases (Chen et al., 2015), of which similar approaches have been used for miRNA-miRNA network inference (Xu et al., 2013a). The lncRNA similarities that we used are calculated by Chen's method. For instance, the lncRNA *l*_*a*_ is associated with a group of diseases *DT*_*a*_ = {*d*_*i*1_, *d*_*i*2_, …, *d*_*im*_}, lncRNA *l*_*b*_ is associated with a group of diseases *DT*_*b*_ = {*d*_*j*1_, *d*_*j*2_, …, *d*_*jn*_}. The similarity between *DT*_*a*_ and *DT*_*b*_ is then calculated as the similarity of *l*_*a*_ and *l*_*b*_, and it is denoted as *LS*(*l*_*a*_, *l*_*b*_). *LS*(*l*_*a*_, *l*_*b*_) is defined as,

(1) $$\begin{array}{l} LS\left(l_{a},l_{b}\right)=\frac{\sum_{i=1}^{m}\underset{1\leq j\leq n}{\max}\left(DS\left(d_{ai},d_{bj}\right)\right)+\sum_{j=1}^{n}\underset{1\leq i\leq m}{\max}\left(DS\left(d_{bj},d_{ai}\right)\right)}{m+n}, \end{array}$$

where *DS*(*d*_*ai*_, *d*_*bj*_) is the semantic similarity of disease of *d*_*ai*_ and *d*_*bj*_ which belong to *DT*_*a*_ and *DT*_*b*_ respectively. *m* and *n* are the numbers of diseases that are included by *DT*_*a*_ and *DT*_*b*_. The lncRNA similarities are denoted by matrix $L\epsilon {\mathbb{R}}^{n_{l}\times n_{l}}$ where *L*_*ij*_ is the similarity of two lncRNAs *l*_*i*_ and *l*_*j*_ (Figure 1A).

#### Representation of the lncRNA-miRNA Interactions

It is well-known that the lncRNAs often interact with the corresponding miRNAs and they are involved in the biological processes synchronously (Yang et al., 2014; Paraskevopoulou and Hatzigeorgiou, 2016). Hence our prediction model also takes the interaction relationships between lncRNAs and miRNAs into account (Figure 1C). The interactions between *n*_*l*_ lncRNAs and *n*_*m*_ miRNAs are represented by the matrix $Y\epsilon {\mathbb{R}}^{n_{l}\times n_{m}}$, and each row of ***Y*** corresponds to a lncRNA and each column of ***Y*** corresponds to a miRNA. *Y*_*ij*_ is 1 when lncRNA *l*_*i*_ interacts with miRNA *m*_*j*_ and it is 0 otherwise.

### Disease lncRNA Prediction Model Based on Dual CNN

**Figure 2:**
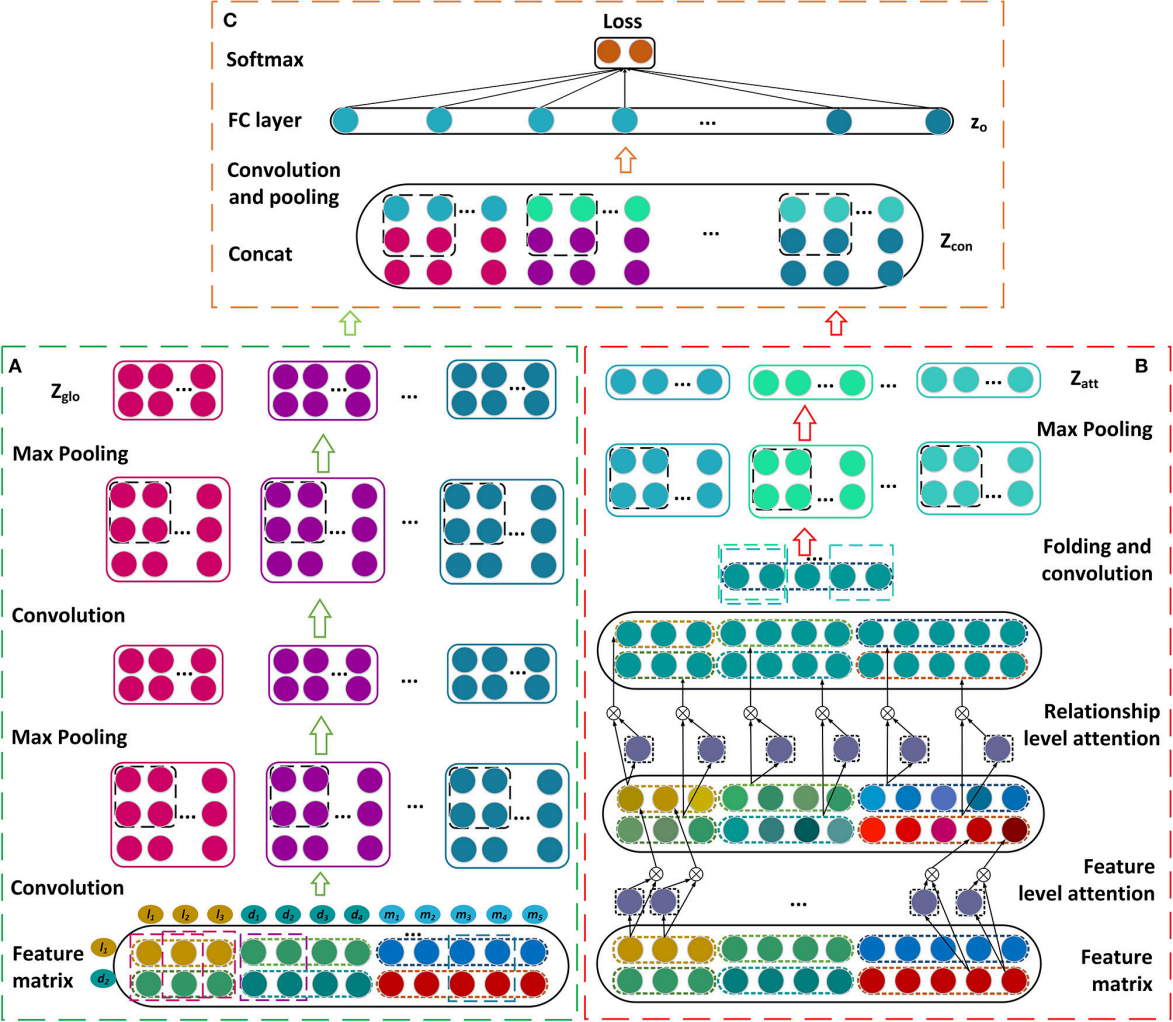
Construction of the framework based on the dual convolutional neural networks for learning the global and attention representations. **(A)** Construct the convolutional and pooling layers. **(B)** Establish the attention mechanism at the feature and relationship levels. **(C)** Construct the final module to estimate the association score.

In this section, we describe our prediction model for learning the latent representations of lncRNA-disease associations and predicting the disease-related lncRNAs. The feature matrix is constructed firstly by incorporating the similarities, interactions, and associations about lncRNAs, miRNAs, and diseases (Figure 1). A novel framework is then established based on dual convolutional neural networks with attention mechanisms (Figure 2). The left part of the framework learns the global representation of a lncRNA-disease association, while the right part learns the more informative connection relationships among lncRNAs, miRNAs, and diseases. These two representations are integrated by an additional convolutional and fully connected layer and the possibility that a lncRNA is associated with a disease is obtained as their association score. We take the lncRNA *l*_1_ and the disease *d*_2_ as an example to describe our model CNNLDA for lncRNA-disease association prediction.

#### Construction of Feature Matrix

The feature matrix of the lncRNA *l*_1_ and the disease *d*_2_ is constructed by combining three biological premises. First, if *l*_1_ and *d*_2_ have similarity and association relationships with more common lncRNAs, they are more likely associated with each other. For instance, if *l*_1_ and *l*_2_ have similar functions, and *d*_2_ has been observed to be associated with *l*_2_, *l*_1_ will be possibly associated with *d*_2_. Let ***x***_1_ represent the 1st row of ***L*** which contains the similarities between *l*_1_ and the various lncRNAs. The 2nd column of ***D***, ***x***_2_, records the associations between *d*_2_ and all the lncRNAs. ***x***_1_ and ***x***_2_ are put together to form a matrix whose dimension is 2 × *n*_*l*_ (Figure 1A). Second, when *l*_1_ and *d*_2_ have the association and similarity connections with more common diseases, *l*_1_ is more likely to be associated with *d*_2_. ***x***_3_ is the 1st row of ***A*** and it records the associations between *l*_1_ and all the diseases. ***x***_4_ is the 2nd row of ***D*** and it contains the similarities between *d*_2_ and these diseases. ***x***_3_ and ***x***_4_ are also combined and they form a matrix with dimension 2 × *n*_*d*_ (Figure 1B). Third, there is a possible association between *l*_1_ and *d*_2_ when they have the interaction and association connections with the common miRNAs. The 1st row of ***Y***, ***x***_5_, records the interactions between *l*_1_ and the various miRNAs, while the 2nd column of ***B***, ***x***_6_, records the associations between *d*_2_ and these miRNAs. ***x***_5_ and ***x***_6_ are integrated to form a matrix with dimension 2 × *n*_*m*_ (Figure 1C). All of these three matrices are concatenated and then form a feature matrix of lncRNA *l*_1_ and disease *d*_2_ whose dimension is 2 × (*n*_*l*_ + *n*_*d*_ + *n*_*m*_) (Figure 1D).

#### Convolutional Module on the Left

The feature matrix of *l*_1_ and *d*_2_, ***P***, is input to the convolutional module on the left to learn a global deep representation for *l*_1_ and *d*_2_. The convolutional module includes two convolutional layers and two pooling layers (Figure 2A), we take the first convolutional layer and the first pooling layer as examples to describe the process of the convolution and the pooling. To learn the marginal information of ***P***, we pad zeros around ***P*** and obtain a new matrix named ***P***′.

##### Convolutional layer

For the first convolutional layer, the length of a filter is set as *n*_*f*_, and its width is *n*_*w*_. If the number of filters is *n*_*conv*1_, the filters $W_{conv1}\in {\mathbb{R}}^{n_{conv1}\times {n_{w}\times n}_{f}}$ are applied to the matrix ***P***′, and get the feature maps $Z_{conv1}\in {\mathbb{R}}^{n_{conv1}\;\times \left(4-n_{w}+1\right)\times \left(n_{t}+2-n_{f}+1\right)}$. ***P***′(*i, j*) is the element at the *i*th row and the *j*th column of ***P***′, and ***P***′_*k,i,j*_ represents a region within the filter when the *k*th filter slides to the position ***P***′(*i, j*). The formal definitions of ***P***′_*k,i,j*_ and ***Z***_*conv*1,*k*_ are as follows,

(2) $$\begin{array}{l} P\prime_{k,i,j}=P\prime \left(i:i+n_{w},j:j+n_{f}\right),\;P\prime_{k,i,j}\in {\mathbb{R}}^{n_{w}\times n_{f}}, \end{array}$$

(3) $$\begin{array}{l} Z_{conv1,k}\left(i,j\right)=\;f\left(W_{conv1}\left(k,:,:\right)*P\prime_{k,i,j}+b_{conv1}\left(k\right)\right), \end{array}$$

$$\begin{array}{l} i\in \left[1,4-n_{w}+1\right],j\in \left[1,n_{t}+2-n_{f}+1\right],k\in \left[1,n_{conv1}\right], \end{array}$$

where ***b***_*conv*1_ is the bias vector, *f* is a *relu* function (Nair and Hinton, 2010), and *n*_*t*_ = *n*_*l*_ + *n*_*d*_ + *n*_*m*_. ***Z***_*conv*1,*k*_(*i, j*) is the element at the *i*th row and *j*th column of the *k*th feature map ***Z***_*conv*1,*k*_.

##### Pooling layer

We apply the max pooling to extract the robust features from the feature maps ***Z***_*conv*1_. *n*_*g*_ and *n*_*p*_ are the length and width of a filter of pooling layer, respectively. The pooling outputs of all the feature maps are ***Z***_*convpool*1_,

(4) $$\begin{array}{l} Z_{convpool1,k}\left(i,j\right)=Max\left(Z_{conv1,k}\left(i:i+n_{g},j:j+n_{p}\right)\right), \end{array}$$

$$\begin{array}{l} i\in \left[1,5-n_{w}-n_{g}+1\right],j\in \left[1,n_{t}+3-n_{f}-n_{p}+1\right],\;\\ k\in \left[1,n_{conv1}\;\right], \end{array}$$

where ***Z***_*convpool*1,*k*_ is the *k*th feature map, and ***Z***_*convpool*1,*k*_(*i, j*) is the element at its' *i*th row and *j*th column.

#### Attention Module on the Right

In our model, the attention module are used to learn which features or connection relationships are more informative for the representation of lncRNA *l*_1_ and disease *d*_2_. Thus, the module consists of the attention mechanism at the feature level and the one at relationship level (Figure 2B).

##### Attention at the feature level

The features within ***P*** usually have different contributions for representations of lncRNA-disease associations. For instance, in terms of a specific disease, the lncRNAs that have been observed to be associated with the disease are often more important than the unobserved ones. In the feature matrix ***P*** = {***x***_1_, ***x***_2_, …, ***x***_*i*_, …, ***x***_6_ }, each feature *x*_*ij*_ of vector ***x***_*i*_ is assigned an attention weight $\alpha_{ij}^{F}$. $\alpha_{ij}^{F}$ is defined as follows,

(5) $$\begin{array}{l} s_{i}^{F}=H^{F}\tanh\left(W_{x}^{F}x_{i}+b^{F}\right), \end{array}$$

(6) $$\begin{array}{l} \alpha_{ij}^{F}=\;\frac{\exp\left(s_{ij}^{F}\right)}{\sum_{k}\exp\left(s_{ik}^{F}\right)}\;, \end{array}$$

where ***H***^*F*^ and $W_{x}^{F}$are the weight matrices, and ***b***^*F*^ is a bias vector. $s_{i}^{F}=\left[s_{i1}^{F},s_{i2}^{F},\ldots ,s_{ik}^{F},\ldots ,s_{in_{i}}^{F}\right]$ is the vector that records the attention scores representing the importance of different features in ***x***_*i*_, where *n*_*i*_ is the length of ***x***_*i*_, $s_{in_{i}}^{F}$ is the score of *x*_*i*_*n*__*i*__. $\alpha_{ij}^{F}$ is the normalized attention weight for feature *x*_*ij*_. Thus the latent representation of different features may be denoted as ***y***_*i*_,

(7) $$\begin{array}{l} y_{i}=\alpha_{i}^{F}⊗x_{i}\;, \end{array}$$

where ⊗ is the element-wise product operator, and the symbol *F* represents the feature level.

##### Attention at the relationship level

There are several connection relationships among lncRNAs, diseases, and miRNAs, including the similarities between lncRNAs, the associations between lncRNAs and diseases, the similarities between diseases, the interactions between lncRNAs and miRNAs, and the associations between diseases and miRNAs. Different relationships also have different contributions to the representation of lncRNA-disease associations. Therefore, in relationship level, we use an attention mechanism on each feature vector ***y***_*i*_ to generate the final attention representation. The attention scores at relationship level are given by,

(8) $$\begin{array}{l} s_{i}^{R}=h^{R}\tanh\left(W_{y}^{R}y_{i}+b^{R}\right), \end{array}$$

(9) $$\begin{array}{l} \beta_{i}^{R}=\;\frac{\exp\left(s_{i}^{R}\right)}{\sum_{j\in 6}\exp\left(s_{j}^{R}\right)}\;, \end{array}$$

where $W_{y}^{R}$ is the weight matrix, and ***b***^*R*^ is a bias vector. ***h***^*R*^ is a weight vector and $s_{i}^{R}$ represents the score of the *i*th relationship ***y***_*i*_. $\beta_{i}^{R}$ is the normalized attention weight for relationship ***y***_*i*_. The latent representation of association through the attentions at the feature and relationship levels is obtained and represented by

(10) $$\begin{array}{l} g=\sum_{i}\beta_{i}^{R}y_{i}\;, \end{array}$$

where the symbol *R* represents the relationship level. Let ***G*** be the matrix after ***g*** is padding zeros. The attention representations ***Z***_*att*_ are obtained by feeding ***G*** into a convolutional layer and a maxpooling layer.

#### Final Module

Let ***Z***_*glo*_ be the global representation that are learned from the left convolutional module and ***Z***_*att*_ be the attention representation that are learned from the right convolutional module. ***Z***_*glo*_ and ***Z***_*att*_ are combined by putting the former on top and putting the later under it, and denoted as ***Z***_*con*_ (Figure 2C). ***Z***_*con*_ runs through an additional convolutional layer to obtain the final representation ***Z***_*fin*_. ***z***_*o*_ is a vector of flattening ***Z***_*fin*_ and it is inputted into a fully connected layer ***W***_*out*_ and a *softmax* layer (Bahdanau et al., 2014) to get ***p***

(11) $$\begin{array}{l} p=softmax\left(W_{out}z_{o}+b_{o}\right). \end{array}$$

***p*** is an association probability distribution of *C* classes (*C* = 2), and it contains the probability that a lncRNA and a disease is determined to have an association relationship and the probability that they have no association.

#### Loss of Association Prediction

In our model, the cross-entropy loss between the ground truth distribution of lncRNA-disease association and the prediction probability ***p*** is defined as L,

(12) $$\begin{array}{l} L=-\sum_{i}^{T}\sum_{j=1}^{C}z_{j}\log p_{j}, \end{array}$$

where ***z*** ∈ ℝ^2^ is the classification label vector and *T* is a set of training samples. If *l*_1_ is associated with *d*_2_, the second dimension of the vector ***z*** is 1 and the first one is 0. On the contrary, if *l*_1_ is not associated with *d*_2_, the first dimension of ***z*** is 1 and the second one is 0.

We denote all neural network parameters by θ. The objective function in our learning process is defined as follows,

(13) $$\begin{array}{l} \begin{array}{c} \min\\ \theta \end{array}ℒ\left(\theta \right)=ℒ+\lambda {\left\|\theta \right\|}^{2}, \end{array}$$

where λ is a trade-off parameter between the training loss and regularization term. We use Adam optimization algorithm to optimize the objective function (Kingma and Ba, 2015).

## Results and Discussion

### Parameter Setting

In CNNLDA, 2 × 2 window size is used for all of the convolutional and pooling layers. In the left convolutional module (Figure 2A), the number of filters in the first convolutional layer is 8 and one in the second layer is 16. In the right attention convolutional module (Figure 2B), the number of filters is 16. In the final module (Figure 2C), we set the number of filters to 32. We implement our method using Pytorch to train and optimize the neural networks, and a GPU card (Nvidia GeForce GTX 1080Ti) is utilized to speed up the training process. The training process is terminated when the maximum number of iterations, 80, is reached.

### Performance Evaluation Metrics

Five-fold cross-validation is performed to evaluate the performance of CNNLDA and other state-of-the-art methods for predicting lncRNA-disease associations. If a lncRNA *l*_*s*_ is associated with a disease *d*_*t*_, we treat the *l*_*s*_-*d*_*t*_ node pair as a positive sample. If *l*_*s*_ is not observed to associate with *d*_*t*_, it is treated as a negative sample. For each cross validation, we randomly select 80% positive samples and the same number of negative samples as the training data and use the remaining 20% positive samples and all of the negative samples for testing. Note that the association dataset is separated to 5 folds for cross-validation, and we recomputed the lncRNA similarities by using the known associations that are used for training in each cross validation process.

The samples are ranked by their association scores after the association probabilities of the testing samples are estimated. The higher the node pairs of the positive samples are ranked, the better CNNLDA performs. If an observed association exists in lncRNA-disease node pair samples, and its association score is greater than a threshold θ, it is a successfully determined positive sample. If the prediction score of a negative sample is smaller than θ, it is a determined correctly negative sample. We calculate the true positive rates (*TPR*s) and the false positive rates (*FPR*s) to get a receiver operating characteristic (ROC) curve by changing threshold θ. *TPR* and *FPR* are defined as follows,

(14) $$\begin{array}{l} TPR=\frac{TP}{TP+FN}\;,FPR=\frac{FP}{FP+TN}\;, \end{array}$$

where *TP* is the number of successfully identified positive samples, and *FN* is the number of misidentified negative samples. *TN* is the number of correctly identified negative samples, and *FP* is the number of incorrectly identified positive samples. The global prediction performance of a method is always measured by the area under the ROC curve (AUC) (Karimollah, 2013).

The known lncRNA-disease associations (the positive samples) and the unobserved ones (the negative samples) form the serious imbalance. In such case, we also use the precision-recall (PR) curve and its area (AUPR) to assess the performance of a prediction method (Takaya and Marc, 2015). Precision and recall are defined as follows,

(15) $$\begin{array}{l} Precision=\frac{TP}{TP+FP}\;,Recall=\frac{TP}{TP+FN}\;. \end{array}$$

Precision is the rate of the correctly identified positive samples among the samples that are retrieved, and recall is the rate of the correctly identified positive samples among all the positive samples. In terms of 5-fold cross-validation, we use averaging CV to obtain the final performance. Averaging CV means that we obtain a separate performance (AUC or AUPR) for each of the 5 folds when used as a test set, and the 5 performances are averaged to give the final performance.

In addition, the biologists usually select lncRNA candidates from the top part of the ranking list, and then further validate their associations with diseases. Therefore, the recall values of top 30, 60, …, 240, are calculated, and they represent the fraction of the successfully recovered positive samples in the top list *k* among the total positive samples.

### Comparison With Other Methods

**Figure 3:**
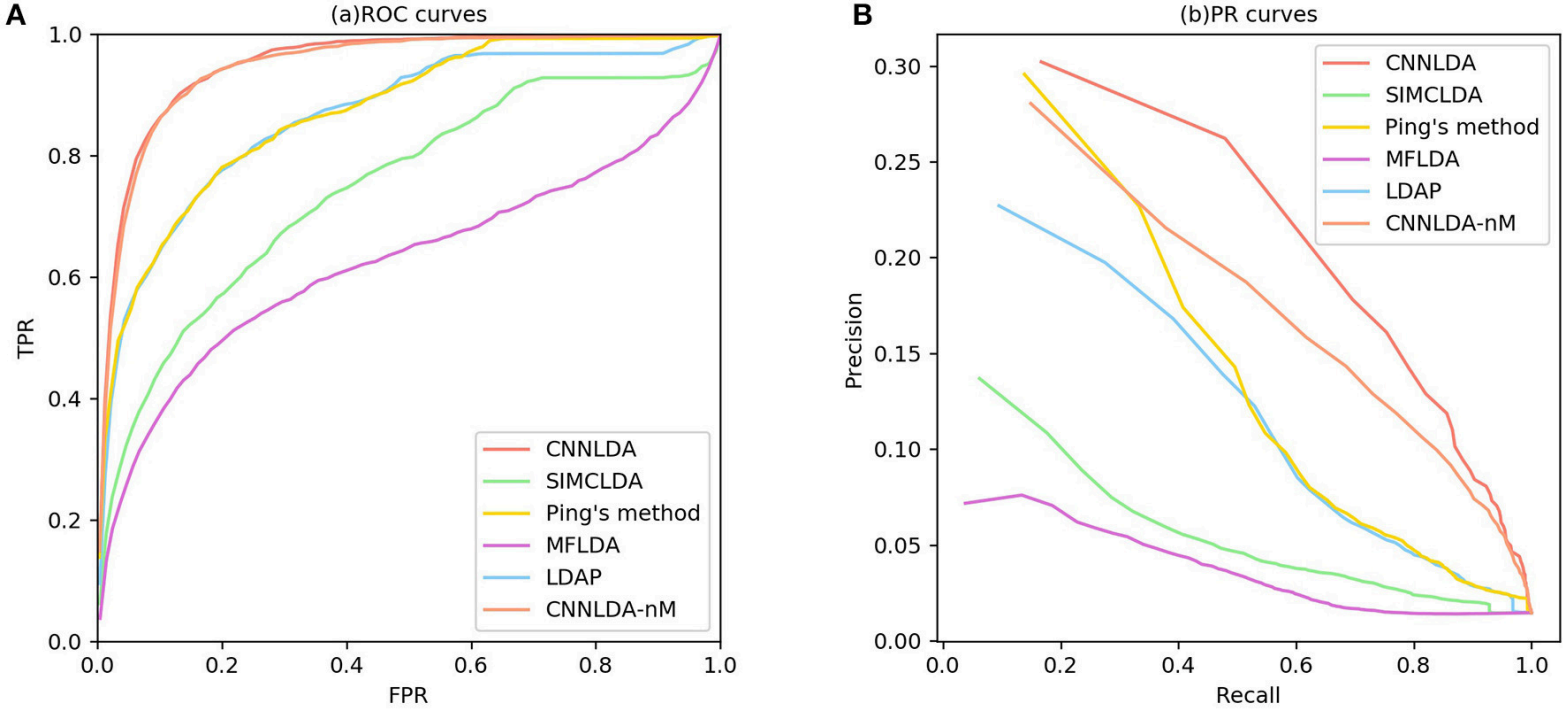
ROC curves and PR curves of CNNLDA and other methods for all the diseases. **(A)** ROC curves of all the methods. **(B)** PR curves of all the methods.

To evaluate the performance of CNNLDA, we compare it with several state-of-the-art methods including SIMCLDA (Lu et al., 2018), Ping's method (Ping et al., 2018), MFLDA (Fu et al., 2017) and LDAP (Lan et al., 2017) for lncRNA-disease association prediction. As shown in Figure 3A and Table 1, CNNLDA achieves the highest average AUC on all of the tested 402 diseases (AUC = 0.952). It outperforms SIMCLDA by 20.6%, Ping's method by 8.05%, MFLDA by 32.6% and LDAP by 8.85%. We also list the AUCs of the five methods on 10 well-characterized diseases that are associated with at least 15 lncRNAs (Table 1). CNNLDA yields the best performance for 9 out of 10 diseases. CNNLDA achieves best average performance (AUPR = 0.251) which is 15.6%, 3.19, 18.5, and 8.51% better than SIMCLDA, Ping's method, MFLDA and LDAP respectively (Figure 3B). In addition, CNNLDA achieves the highest AUPRs on 9 out of 10 well-characterized diseases (Table 2). The performance of Ping's method is similar to that of LDAP as they exploit different types of similarities of lncRNAs and diseases. These two methods achieves the second and third best performance respectively. The performance of MFLDA is not as good as the other four methods as it did not exploit the disease similarities and the lncRNA similarities. The improvement of CNNLDA over the compared methods is primarily due to its deeply learning the global and attention representations of lncRNA-disease associations.

**Table 1:** AUCs of ROC curves of CNNLDA and other methods for all of the diseases and 10 well-characterized diseases.

**Disease name**	**AUC of ROC curve**				
	**CNNLDA**	**SIMCLDA**	**Ping's method**	**MFLDA**	**LDAP**
Average AUC on 402 diseases	**0.952**	0.746	0.871	0.626	0.863
Respiratory system cancer	0.885	0.789	**0.911**	0.719	0.891
Organ system cancer	**0.967**	0.82	0.95	0.729	0.884
Intestinal cancer	**0.955**	0.811	0.909	0.559	0.905
Prostate cancer	**0.897**	0.873	0.826	0.553	0.71
Lung cancer	**0.94**	0.79	0.911	0.676	0.883
Breast cancer	**0.836**	0.742	0.871	0.517	0.83
Reproductive organ cancer	**0.922**	0.707	0.818	0.74	0.742
Gastrointestinal system cancer	**0.945**	0.784	0.896	0.582	0.867
Liver cancer	**0.918**	0.799	0.91	0.634	0.898
Hepatocellular carcinoma	**0.922**	0.765	0.903	0.688	0.902

*The bold values significant the highest AUC*.

**Table 2:** AUPRs of PR curves of CNNLDA and other methods for all of the diseases and 10 well-characterized diseases.

**Disease name**	**AUPR of PR curve**				
	**CNNLDA**	**SIMCLDA**	**Ping's method**	**MFLDA**	**LDAP**
Average AUPR on 402 diseases	**0.251**	0.095	0.219	0.066	0.166
Respiratory system cancer	0.245	0.149	**0.414**	0.072	0.303
Organ system cancer	**0.795**	0.411	0.765	0.338	0.628
Intestinal cancer	**0.406**	0.141	0.252	0.042	0.246
Prostate cancer	**0.390**	0.176	0.333	0.095	0.297
Lung cancer	0.058	0.138	**0.334**	0.008	0.094
Breast cancer	**0.964**	0.445	0.803	0.476	0.629
Reproductive organ cancer	0.091	0.047	**0.403**	0.031	0.396
Gastrointestinal system cancer	**0.441**	0.130	0.271	0.104	0.238
Liver cancer	**0.666**	0.201	0.526	0.086	0.498
Hepatocellular carcinoma	**0.323**	0.096	0.239	0.082	0.303

*The bold values significant the highest AUPR*.

We perform a paired *Wilcoxon*-test to evaluate whether CNNLDA's AUCs and AUPRs across all of the tested diseases are significantly higher than those of another method. CNNLDA achieves significantly higher performance than the other methods in terms of both AUCs and AUPRs as the corresponding *P*-values are smaller than 0.05 (Table 3).

**Table 3:** A pairwise comparison with a paired Wilcoxon-test on the prediction results in terms of AUCs and AUPRs.

***P*-value between CNNLDA and another method**	**SIMCLDA**	**Ping's method**	**MFLDA**	**LDAP**
*P*-values of ROC curves	7.2911e-116	7.7561e-53	1.3120e-133	3.7677e-64
*P*-values of PR curves	1.7468e-41	0.0455	5.0559e-52	4.8014e-09

**Figure 4:**
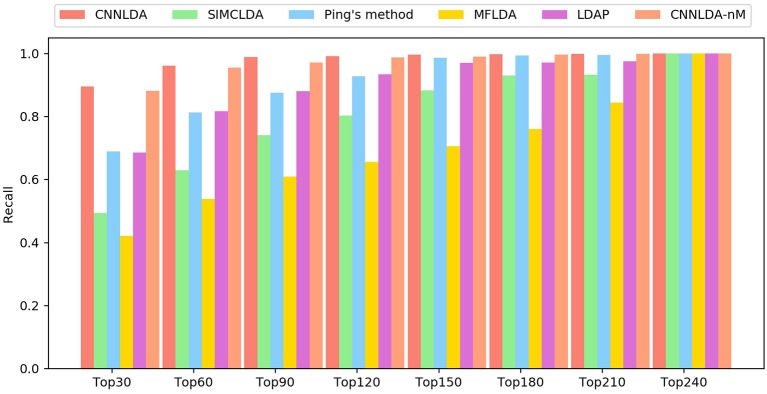
The average recalls across all the tested diseases under different top *k* cutoffs.

The higher the recall rate on the top *k* ranked lncRNA-disease associations is, the more genuine associations are determined correctly. Under different *k* cutoffs, the performance of CNNLDA consistently outperforms other methods (Figure 4), and ranks 89.6% of the positive samples in the top 30, 96.2% in the top 60, and 98.8% in the top 90. Most of the recalls of Ping's method are very close to LDAP, while Ping's method ranks 68.9% in top 30, 81.3% in top 60, 88% in top 90. LDAP ranks 68.5% in top 30, 81.3% in top 60, 88% in top 90. SIMCLDA ranks 49.3% in top 30, 63% in top 60, 74.1% in top 90, which is not as good as Ping's method but better than MFLDA (42%, 53.9% and 61%).

In addition, to validate the effectiveness of exploiting the information related to the miRNAs, we construct another instance of CNNLDA that is trained without this kind of information, and the instance is referred to as CNNLDA-nM. The instance of CNNLDA that is trained by using the miRNA-related information is still named as CNNLDA. CNNLDA's AUC and AUPR are 0.2%and 0.94% greater than CNNLDA-nM, which confirms the importance of integrating the information for improving CNNLDA's prediction performance.

### Case Studies: Stomach Cancer, Lung Cancer, and Colon Cancer

To demonstrate CNNLDA's ability to discover potential candidate disease lncRNAs, we execute the case studies on *stomach cancer, lung cancer*, and *colon cancer* and analyze the top 15 candidates respectively related to these cancers (Table 4).

**Table 4:** The candidate lncRNAs associated with stomach cancer, lung cancer and colon cancer.

**Disease name**	**Rank**	**LncRNA name**	**Description**	**Rank**	**LncRNA name**	**Description**
Stomachcancer	1	XIST	LncRNADisease, Lnc2Cancer	9	HULC	LncRNADisease, Lnc2Cancer
	2	NEAT1	LncRNADisease, Lnc2Cancer	10	PCAT1	Lnc2Cancer
	3	SOX2-OT	Lnc2Cancer	11	HOTTIP	LncRNADisease, Lnc2Cancer
	4	CCAT2	LncRNADisease, Lnc2Cancer	12	KCNQ1OT1	literature^1^ Sun et al., 2018
	5	TUG1	LncRNADisease, Lnc2Cancer	13	WT1-AS	LncRNADisease, Lnc2Cancer
	6	MALAT1	LncRNADisease, Lnc2Cancer	14	NPTN-IT1	miRCancer, StarBase
	7	BCYRN1	Lnc2Cancer	15	MIR17HG	literature^1^ Bahari et al., 2015
	8	HCP5	literature^2^ Mo et al., 2018			
Lung cancer	1	HOTTIP	LncRNADisease, Lnc2Cancer	9	LINC00663	Lnc2Cancer
	2	PCA3	unconfirmed	10	SOX2-OT	LncRNADisease
	3	LINC00675	unconfirmed	11	MIAT	Lnc2Cancer
	4	HULC	literature^1^Zhang et al., 2016	12	LINC00312	Lnc2Cancer
	5	KCNQ1OT1	Lnc2Cancer	13	TINCR	Lnc2Cancer
	6	SNHG12	Lnc2Cancer	14	LINC00961	Lnc2Cancer
	7	CBR3-AS1	miRCancer, StarBase	15	GHET1	Lnc2Cancer
	8	TUSC7	Lnc2Cancer			
Colon cancer	1	PVT1	Lnc2Cancer	9	SNHG4	miRCancer, StarBase
	2	UCA1	LncRNADisease, Lnc2Cancer	10	SPRY4-IT1	literature^1^ Shen et al., 2017
	3	NEAT1	Lnc2Cancer	11	BANCR	Lnc2Cancer
	4	WT1-AS	Lnc2Cancer	12	HULC	Lnc2Cancer
	5	CDKN2B-AS1	Lnc2Cancer	13	LSINCT5	Lnc2Cancer
	6	BCYRN1	literature^1^ Gu et al., 2018	14	KCNQ1OT1	Lnc2Cancer
	7	GAS5	Lnc2Cancer	15	HNF1A-AS1	Lnc2Cancer
	8	HOTAIRM1	Lnc2Cancer			

*(1) “Lnc2Cancer” and “LncRNADisease” are manually curated database. (2) “literature^1^” means that published literature supports that dysregulation of the lncRNA in cancer. (3) “literature^2^”or “miRCancer, StarBase” means that the lncRNA is related to some important factors affecting the development of the cancer*.

First, a database named Lnc2Cancer curates the lncRNAs that have different expression in the disease tissues compared to the normal ones. Lnc2Cancer contains lncRNAs related to cancers that have been identified by analyzing the results of northern blot experiments, microarray experiments, and quantitative real-time polymerase chain reaction experiments (Gao et al., 2018). LncRNADisease is also a database which includes 2,947 lncRNA-disease entries (Chen et al., 2013). By using text mining techniques, these associations are extracted from the published literature, and then the dysregulation of lncRNAs are manually confirmed. As shown in Table 4, 33 candidate lncRNAs are contained by Lnc2Cancer and 13 candidate lncRNAs are included by LncRNADisease, which confirms these lncRNAs have been upregulated or downregulated in these cancers.

Next, 2 candidates of stomach cancer, 1 candidate of lung cancer and 2 candidates of colon cancer labeled with “literature^1^” are supported by several published literature. These lncRNAs are confirmed to have dysregulations in the cancers when compared with the normal tissues (Bahari et al., 2015; Zhang et al., 2016; Shen et al., 2017; Gu et al., 2018; Sun et al., 2018).

Finally, 5 candidates labeled with “literature^2^,” and “miRCancer, StarBase” are related to the important factors affecting the development of the corresponding cancers. In the metabolic network, lncRNA HCP5 is regulated by three miRNAs, and the miRNAs are downregulated in stomach cancer. It indicates that the expression of HCP5 is more likely to associate with stomach cancer (Mo et al., 2018). Four lncRNAs (CBR3-AS1, NPTN-IT1, CDKN2B-AS1 and SNHG4) have interactions with four corresponding miRNAs (hsa-miR-217, hsa-miR-520c-3p, hsa-miR-320a and hsa-miR-4458) (Li et al., 2014b). These four miRNAs have been to be observed associated with stomach cancer, lung cancer and colon cancer (Xie et al., 2013). Hence these lncRNAs are probably involved in the progression of these cancers.

### Predicting Novel Disease-Related lncRNAs

After evaluating its prediction performance through the cross-validation process and case studies, CNNLDA is applied to all 402 diseases. All the positive samples and the negative ones are used to train CNNLDA to predict the novel disease-associated lncRNAs. The potential candidate lncRNAs for these diseases are listed in Supplementary Table 1. In addition, the lncRNA similarities based on the diseases associated with these lncRNAs are shown in Supplementary Table 2.

## Conclusions

A novel method based on dual convolutional neural networks, CNNLDA, is developed for predicting the potential disease-related lncRNAs. We respectively construct the attention mechanism at feature and relationship levels to discriminate the different contributions of features and learn the more informative representation of lncRNA-disease associations. The new framework based on dual convolutional neural networks is developed for learning the global representation and the attention of lncRNA-disease associations. The experimental results indicate that CNNLDA is superior to the compared other methods in terms of both AUCs and AUPRs. The case studies on 3 diseases demonstrate CNNLDA's ability for discovering potential disease-associated lncRNAs.

## Data Availability

All datasets analyzed for this study are cited in the manuscript and the Supplementary Files.

## Author Contributions

PX and YC conceived the prediction method, and they wrote the paper. YC and ZZ developed computer programs. TZ and RK analyzed the results and revised the paper.

### Conflict of Interest Statement

The authors declare that the research was conducted in the absence of any commercial or financial relationships that could be construed as a potential conflict of interest.

## Supplementary Material

Supplementary Table 1 Potential candidate lncRNAs related to 402 diseases.

Supplementary Table 2 The lncRNA similarities.
